# The SWEET gene family in *Hevea brasiliensis* – its evolution and expression compared with four other plant species

**DOI:** 10.1002/2211-5463.12332

**Published:** 2017-10-30

**Authors:** Jin‐Lei Sui, Xiao‐Hu Xiao, Ji‐Yan Qi, Yong‐Jun Fang, Chao‐Rong Tang

**Affiliations:** ^1^ Institute of Tropical Agriculture and Forestry Hainan University Haikou Hainan China; ^2^ Key Lab of Rubber Biology Ministry of Agriculture & Rubber Research Institute Chinese Academy of Tropical Agricultural Sciences Danzhou Hainan China

**Keywords:** gene expression, *Hevea brasiliensis*, structure and evolution, sugar transport, SWEET

## Abstract

SWEET proteins play an indispensable role as a sugar efflux transporter in plant development and stress responses. The *SWEET* genes have previously been characterized in several plants. Here, we present a comprehensive analysis of this gene family in the rubber tree, *Hevea brasiliensis*. There are 36 members of the SWEET gene family in this species, making it one of the largest families in plant genomes sequenced so far. Structure and phylogeny analyses of these genes in Hevea and in other species demonstrated broad evolutionary conservation. RNA‐seq analyses revealed that *SWEET2, 16,* and *17* might represent the main evolutionary direction of *SWEET* genes in plants. Our results in Hevea suggested the involvement of *HbSWEET1a*,* 2e*,* 2f*, and *3b* in phloem loading, *HbSWEET10a* and *16b* in laticifer sugar transport*,* and *HbSWEET9a* in nectary‐specific sugar transport. Parallel studies of RNA‐seq analyses extended to three other plant species (*Manihot esculenta*,* Populus trichocarpa*, and *Arabidopsis thaliana*) produced findings which implicated *MeSWEET10a, 3a,* and *15b* in *M. esculenta* storage root development, and the involvement of *PtSWEET16b* and *PtSWEET16d* in *P. trichocarpa* xylem development. RT‐qPCR results further revealed that *HbSWEET10a, 16b,* and *1a* play important roles in phloem sugar transport. The results from this study provide a foundation not only for further investigation into the functionality of the SWEET gene family in Hevea, especially in its sugar transport for latex production, but also for related studies of this gene family in the plant kingdom.

**Abbreviations**
ABA: abscisic acid
GSDS: gene structure display server
SRA: sequence read archive
Suc: sucrose

SWEET (Sugars Will Eventually be Exported Transporter) proteins, which feature up to seven transmembrane TM helix domains, selectively transport different kinds of sugar substrates, including sucrose, fructose, and glucose 1. As a sugar efflux transporter, SWEET proteins play important roles in plant growth and development, stress responses, and plant–microbe interactions. Cellular sugar efflux is an essential function in many processes, such as phloem loading, nectar secretion, nourishing symbionts such as mycorrhiza, and in maternal efflux for filial tissue development 1. Sugar efflux systems can be hijacked by pathogens for access to nutrition from hosts 2, and accordingly, mutations that block recruitment of the efflux mechanism by the pathogen facilitate plant resistance to their attack 3. Previous studies on SWEETs have been focused mainly on two model plant species, namely *Arabidopsis thaliana* and *Oryza sativa* 4, 5, 6. In *A. thaliana*, 17 SWEET family members have been characterized, and they fall into four phylogenetic clades, in which AtSWEET1‐3 are in Clade I, AtSWEET4‐8 in Clade II, AtSWEET9‐15 in Clade III, and AtSWEET16‐17 in Clade IV. The SWEETs from most of the other plants are named following the nomenclature adopted in *A. thaliana*. SWEET genes, in their many isoforms, are versatile and their functions in plants are widely encompassing. For example, AtSWEET1 acts as a glucose transporter 7, with AtSWEET9 being a nectary‐specific sugar transporter which is essential for nectar production 6. AtSWEET11 and AtSWEET12 catalyze sucrose export from phloem parenchyma cells in source leaves and play a critical role in phloem loading 5. AtSWEET16 and AtSWEET17, as vacuolar SWEET proteins, function as fructose‐specific exporters, connecting the vacuolar lumen to the cytosol 8, 9. In *O. sativa*, OsSWEET11*,* located on the plasma membrane and expressed in the phloem of leaves, is presumably involved in phloem loading, as is the case with its Arabidopsis homologues, *AtSWEET11* and *AtSWEET12* 5. *OsSWEET11* and *OsSWEET14* are specifically exploited by bacterial pathogens for virulence by means of direct binding of a bacterial effector to the SWEET promoter 4, 10. Recently, genome‐wide expression patterns of SWEET genes have been characterized in *Brassica napus, Pyrus bretschneideri, Sorghum bicolor,* and *Glycine max* 11, 12, 13, 14, all pointing to important roles of SWEETs in plant growth, development, and stress responses.

Natural rubber (*cis*‐1,4‐polyisoprene, NR) is an important industrial and strategic raw material, the sole commercial source of which is *Hevea brasiliensis* (the Para rubber tree, Hevea hereafter), a perennial tropical tree species 15. As sucrose is the precursor molecule for rubber biosynthesis and latex regeneration 16, understanding the mechanisms of its transport and metabolism in the rubber tree is of fundamental importance to improving rubber productivity 17. Significant progress was made in the understanding of Hevea sucrose transport and metabolism with the cloning of six sucrose transporter (SUT) genes, among which *HbSUT3* (*HbSUT1B*) was found to be the key member responsible for sucrose loading into laticifers 18, 19. *HbNIN2* has also been identified as the key gene responsible for sucrose catabolism in rubber‐producing laticifers 20. Moreover, two Hevea sucrose synthase genes, *HbSUS2* and *HbSUS3*, were found to exert negative control over sucrose catabolism in the laticifers 21. Hevea *SWEET* genes have not hitherto been investigated in detail, but their characterization has recently been facilitated by the Hevea genome and transcriptome having been independently sequenced by research groups from China 22, Malaysia 23, 24, and Thailand 25, and by the availability of the proteome of Hevea latex 26.

We report here a genome‐wide analysis of the SWEET gene family in Hevea where we compare the results with those from four other plant species, *viz. Manihot esculenta* and *Ricinus communis* belonging to the same family (Euphorbiaceae) as Hevea, and two model plants, *A. thaliana* and *Populus trichocarpa*. The study encompassed a total of 127 *SWEET* genes, the expression patterns of which were analyzed in different tissues in response to various treatments, and at several phases of tissue development. In addition, the gene structure and phylogeny of these genes were compared to help further understanding of the roles of *SWEET* genes in Hevea sugar transport. As a further objective in this investigation, data on *SWEET* genes and their expression in four other plant species were examined, along with the results from Hevea, to compare the structure of their respective gene families and appraise the functions of their members in the plant kingdom.

## Results and Discussion

### Genome‐wide identification of SWEET gene families in Hevea and four other plant species

We identified all *SWEET* gene family members in five plant species (Hevea*, A. thaliana, P. trichocarpa, M. esculenta,* and *R. communis*) from their published genome sequences. In this exercise, the *SWEET* genes from the three *Euphorbiaceae* plants (Hevea*, M. esculenta,* and *R. Communis*) were characterized for the first time. The most recent genome and protein sequences of these species were downloaded from Phytozome v10. Local BLAST searches of the genomes were performed using the published *SWEET* sequences of three model plants of *A. thaliana*,* O. sativa,* and *P. trichocarpa* as queries 1, 5. This analysis identified a total of 127 *SWEET* genes in the five selected plant species, comprising 36 *SWEET* genes in Hevea (Table 1a) 22, 28 in *M. esculenta* (Table 1b)*,* 18 in *R. communis* (Table 1c), 17 in *A. thaliana* 10, and 28 in *P. trichocarpa* 6. All the SWEET gene members newly identified this study were named according to the nomenclature of the *A. thaliana SWEET* gene family. The gene numbers of *SWEET* families identified here for the two model plants (*A. thaliana* and *P. trichocarpa*) matched those previously reported 5.

**Table 1:** Characteristics of SWEET genes in three *Euphorbiaceae* members, *H. brasiliensis, M. esculenta,* and *R. communis*

SWEET*s*	ID	CDS length in bp	Predicted protein			No. of introns	Group
			Length (aa)	Isoelectric point	Mol Wt		
(A) *H. brasiliensis*							
*HbSWEET1a*	scaffold1368_1746	753	251	10.09	27668.01	5	Clade I
*HbSWEET1b*	scaffold4412_5699	750	250	8.82	27885.95	5	Clade I
*HbSWEET1c*	scaffold2014_38474	747	249	9.81	27486.73	5	Clade I
*HbSWEET2a*	scaffold0633_726258	705	235	8.81	26322.47	5	Clade I
*HbSWEET2b*	scaffold0291_2393	606	202	8.58	22646.22	4	Clade I
*HbSWEET2c*	scaffold0649_515754	591	197	9.54	21862.14	4	Clade I
*HbSWEET2d*	scaffold1397_73855	543	181	9.98	20036.78	5	Clade I
*HbSWEET2e*	scaffold0991_115099	705	235	8.53	25981.78	5	Clade I
*HbSWEET2f*	scaffold1207_75820	504	168	8.02	18705.26	3	Clade I
*HbSWEET3a*	scaffold0047_2029699	747	249	9.76	27958.14	5	Clade I
*HbSWEET3b*	scaffold0802_319652	747	249	9.93	28196.37	5	Clade I
*HbSWEET4a*	scaffold0250_352964	726	242	8.69	26996.28	5	Clade II
*HbSWEET4b*	scaffold0371_980268	771	257	6.76	28814.09	5	Clade II
*HbSWEET4c*	scaffold0371_939664	561	187	8.89	20630.72	3	Clade II
*HbSWEET5a*	scaffold0121_20098	714	238	9.60	26665.18	5	Clade II
*HbSWEET5b*	scaffold0190_471668	543	181	9.93	20425.77	3	Clade II
*HbSWEET6*	scaffold1545_54737	774	258	10.00	28336.02	4	Clade II
*HbSWEET7*	scaffold1143_36139	783	261	9.96	28805.48	4	Clade II
*HbSWEET9a*	scaffold1512_21440	768	256	10.20	28969.04	5	Clade III
*HbSWEET9b*	scaffold0030_998488	813	271	9.11	30400.98	5	Clade III
*HbSWEET10a*	scaffold1273_165194	684	228	8.44	26296.42	5	Clade III
*HbSWEET10b*	scaffold0491_348730	828	276	9.10	31738.97	5	Clade III
*HbSWEET10c*	scaffold1273_149445	681	227	8.45	25949.84	3	Clade III
*HbSWEET10d*	scaffold0491_383573	783	261	9.35	29770.06	5	Clade III
*HbSWEET10e*	scaffold0462_183492	726	242	10.11	27840.30	4	Clade III
*HbSWEET10f*	scaffold0491_387781	819	273	7.37	31209.06	5	Clade III
*HbSWEET11*	scaffold0807_24959	846	282	8.91	31803.87	5	Clade III
*HbSWEET12*	scaffold0807_8989	846	282	8.65	31780.94	5	Clade III
*HbSWEET15a*	scaffold0177_54016	693	231	7.03	26007.72	3	Clade III
*HbSWEET15b*	scaffold0868_88200	900	300	6.88	33874.84	5	Clade III
*HbSWEET16a*	scaffold1307_48627	750	250	8.59	27904.85	5	Clade IV
*HbSWEET16b*	scaffold0566_478727	732	244	7.43	26617.46	5	Clade IV
*HbSWEET16c*	scaffold0625_502257	702	234	6.51	25723.25	5	Clade IV
*HbSWEET17a*	scaffold0340_202757	618	206	9.54	22303.57	4	Clade IV
*HbSWEET17b*	scaffold0340_208877	915	305	9.70	33568.20	5	Clade IV
*HbSWEET17c*	scaffold0878_306703	678	226	8.96	24905.58	4	Clade IV
(B) *M. esculenta*							
*MeSWEET1a*	cassava4.1_014638 m	750	250	9.63	27676.81	5	Clade I
*MeSWEET1b*	cassava4.1_014650 m	750	250	9.75	27920.23	5	Clade I
*MeSWEET2a*	cassava4.1_015227 m	702	234	8.55	26066.93	5	Clade I
*MeSWEET2b*	cassava4.1_030719 m	564	188	7.89	20898.87	3	Clade I
*MeSWEET3a*	cassava4.1_026477 m	741	247	9.63	27553.91	5	Clade I
*MeSWEET3b*	cassava4.1_022559 m	672	224	9.61	25566.19	3	Clade I
*MeSWEET4*	cassava4.1_016815 m	582	194	9.37	21364.54	3	Clade II
*MeSWEET5*	cassava4.1_026390 m	714	238	9.09	26997.57	5	Clade II
*MeSWEET6*	cassava4.1_014231 m	780	260	10.12	28528.22	4	Clade II
*MeSWEET7*	cassava4.1_028141 m	777	259	9.98	28488.95	4	Clade II
*MeSWEET9a*	cassava4.1_032222 m	678	226	9.40	25435.76	5	Clade III
*MeSWEET9b*	cassava4.1_031208 m	813	271	8.33	30284.89	5	Clade III
*MeSWEET10a*	cassava4.1_013474 m	840	280	8.18	31795.01	5	Clade III
*MeSWEET10b*	cassava4.1_015602 m	675	225	8.03	25795.72	3	Clade III
*MeSWEET10c*	cassava4.1_021350 m	843	281	9.05	31710.95	5	Clade III
*MeSWEET10d*	cassava4.1_013519 m	837	279	7.81	31755.76	5	Clade III
*MeSWEET10e*	cassava4.1_032927 m	846	282	8.44	31954.92	5	Clade III
*MeSWEET11*	cassava4.1_028116 m	852	284	8.09	31982.66	5	Clade III
*MeSWEET12a*	cassava4.1_017557 m	522	174	6.24	19568.11	2	Clade III
*MeSWEET13*	cassava4.1_026944 m	834	278	8.88	31415.27	5	Clade III
*MeSWEET15a*	cassava4.1_026251 m	717	239	9.63	27175.79	5	Clade III
*MeSWEET15b*	cassava4.1_014124 m	789	263	9.64	29723.69	6	Clade III
*MeSWEET16a*	cassava4.1_014996 m	723	241	8.16	26331.94	5	Clade IV
*MeSWEET16b*	cassava4.1_015143 m	711	237	7.24	25939.66	5	Clade IV
*MeSWEET17*	cassava4.1_014640 m	750	250	9.20	27984.99	5	Clade IV
*MeSWEET17a*	cassava4.1_032999 m	513	171	9.59	18675.24	4	Clade IV
*MeSWEET17b*	cassava4.1_012690 m	906	302	9.83	33200.06	5	Clade IV
*MeSWEET17c*	cassava4.1_014587 m	753	251	9.76	27550.75	5	Clade IV
(C) *R. communis*							
*RcSWEET1*	27985.m000892	744	248	10.08	27412.66	5	Clade I
*RcSWEET2*	30026.m001515	504	168	9.19	18787.52	3	Clade I
*RcSWEET3*	30169.m006529	753	251	9.27	28219.28	5	Clade I
*RcSWEET4a*	29822.m003349	582	194	7.44	21739.97	3	Clade II
*RcSWEET4b*	27613.m000628	699	233	6.64	25779.37	0	Clade II
*RcSWEET4c*	29475.m000237	708	236	7.98	26033.96	0	Clade II
*RcSWEET4d*	29822.m003348	726	242	8.80	27262.76	5	Clade II
*RcSWEET5*	30147.m013970	645	215	9.07	24416.23	3	Clade II
*RcSWEET6*	30068.m002528	783	261	9.98	28738.3	4	Clade II
*RcSWEET9*	29647.m002020	858	286	8.42	32111.69	5	Clade III
*RcSWEET10a*	30147.m014446	831	277	9.05	31790.91	5	Clade III
*RcSWEET10b*	30147.m014447	837	279	9.00	31743.64	5	Clade III
*RcSWEET11*	30147.m014444	855	285	8.04	32313.45	5	Clade III
*RcSWEET12*	30147.m014445	891	297	8.27	33206.31	5	Clade III
*RcSWEET15*	29929.m004599	816	272	10.07	30647.06	6	Clade III
*RcSWEET16a*	29579.m000197	747	249	7.08	27723.79	5	Clade IV
*RcSWEET16b*	29726.m004066	732	244	8.27	26803.66	5	Clade IV
*RcSWEET17*	30128.m008852	864	288	9.95	31371.19	5	Clade IV

The lengths of SWEET‐coding regions (CDS) were similar among the three *Euphorbiaceae* plants examined, ranging from 504 to 915 bp in Hevea, 513 to 906 bp in *M. esculenta*, and 504 to 891 bp in *R. communis* (Table 1). The molecular weights of the SWEET proteins in three *Euphorbiaceae* species ranged from 18.7 to 33.9 kDa, while their isoelectric points (pIs) fell between 6.24 and 10.20 (Table 1).

### Phylogenetic analysis of the SWEET gene families

**Figure 1:**
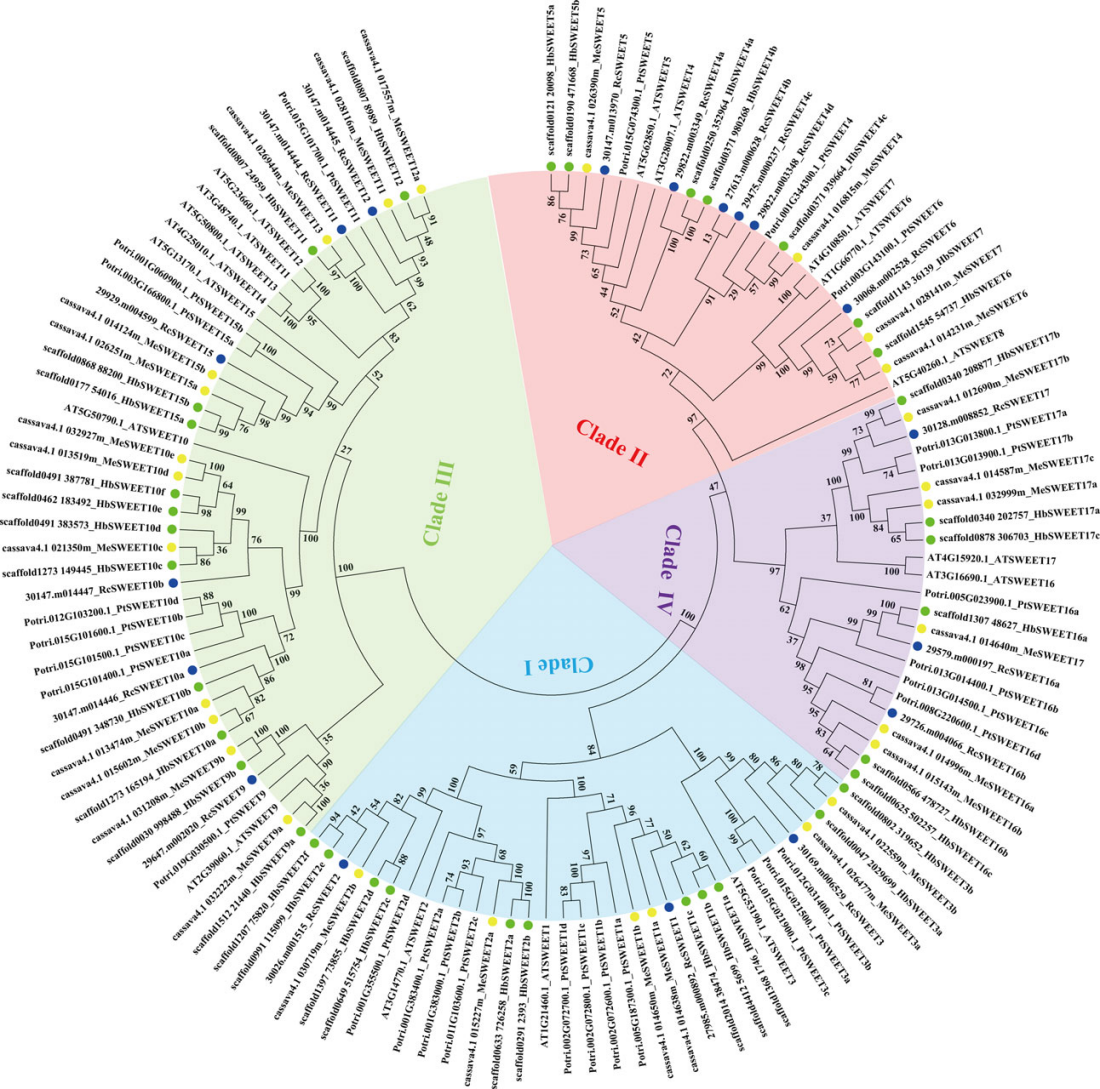
Phylogenetic analysis of the *SWEET* genes in *H. brasiliensis* and four other plant species. An unrooted phylogenetic tree of plant SWEET proteins was constructed using the neighbor‐joining method with the MEGA 6.0 program. Plant species and their SWEET proteins are as follows: *H. brasiliensis*, HbSWEETs (36), marked with green dots; *A. thaliana*, AtSWEETs (17); *P. trichocarpa*, PtSWEETs (28); *M. esculenta*, MeSWEETs (28), marked with yellow dots; *R. communis*, RcSWEETs (18), marked with blue dots.

In order to establish the phylogenetic relationships in the *SWEET* gene families among Hevea and the four other plant species, we aligned the 127 SWEET protein sequences in plants and constructed a phylogenetic tree as shown in Fig. 1 (Table S1). The plant SWEET proteins were clustered into four major groups with high bootstrap values, designated Clades I to IV. The 36 Hevea SWEET genes were dispersed among the four groups with 11, 7, 12, and 6 isoforms, respectively, in Clades I, II, III, and IV. Similarly, the SWEET family of genes in the other four species were also clustered into the above four groups, with 3, 5, 7, and 2 isoforms, respectively, in *A. thaliana*, 11, 3, 8, and 6 in *P. trichocarpa*, 6, 4, 12, and 6 in *M. esculenta*, and 3, 6, 6, and 3 in *R. communis* (Table 1, Fig. 1). Phylogenetic analysis as well as amino acid sequence comparison revealed universal existence of paralogous *SWEET* gene pairs and clusters in the five species. In Hevea, nine such *SWEET* gene pairs (*HbSWEET2a/2b* in Clade I, *HbSWEET2c/2d* in Clade I, *HbSWEET2e/2f* in Clade I, *HbSWEET4a/4b* in Clade II, *HbSWEET5a/5b* in Clade II, *HbSWEET10e/10f* in Clade III, *HbSWEET15a/15b* in Clade III, *HbSWEET16b/16c* in Clade IV, and *HbSWEET17a/17c* in Clade IV) and one gene cluster (*HbSWEET1a, 1b,* and *1c* in Clade I) were identified. Except for the pairs of *HbSWEET2c/2d*,* HbSWEET2e/2f*, and *HbSWEET10e/10f,* the Ka/Ks values of the other paralogous gene pairs were less than 0.5, showing that these genes had undergone a purifying selection (Table 2). The different expression patterns exhibited by the two genes in most of the gene pairs suggested that a functional divergence had occurred after gene duplication (Fig. 4). In *A. thaliana*, there were two *SWEET* gene pairs (*AtSWEET6/7* and *AtSWEET16/17*) and one paralogous gene cluster (*AtSWEET11, 12, 13, 14*). In *P. trichocarpa*, there were two *SWEET* gene pairs (*PtSWEET15a/15b* and *PtSWEET17a/17b*) and four paralogous gene clusters (*PtSWEET1a, 1b, 1c, 1d*;* PtSWEET2a, 2b, 2c*;* PtSWEET3a, 3b, 3c*; and *PtSWEET10a, 10b, 10c, 10d*). In *M. esculenta* and *R. communis,* there was only one *SWEET* gene pair (*MeSWEET10d/10e, RcSWEET4b/4c*) in each species.

**Table 2:** Divergence between paralogous *HbSWEET* gene pairs in *H. brasiliensis*. The synonymous (Ks) and nonsynonymous (Ka) substitution rates between gene duplicate pairs were calculated by KaKs‐Calculator. MA, a model that averages parameters across 14 candidate models

Gene pairs	Method	Ka	Ks	Ka/Ks	*P*‐Value (Fisher)	Length	S‐Sites	N‐Sites
*HbSWEET1b/1c*	MA	0.6391	1.2846	0.4975	4.17E‐17	669	191.017	477.983
*HbSWEET2a/2b*	MA	0.6391	1.2846	0.4975	4.17E‐17	669	191.017	477.983
*HbSWEET2c/2d*	MA	0.1047	0.1065	0.9835	0.8669	543	137.643	405.357
*HbSWEET2e/2f*	MA	0.0080	0.0085	0.9479	0.5795	504	98.1862	405.814
*HbSWEET4a/4b*	MA	0.1419	0.4445	0.3192	4.48E‐12	699	193.225	505.775
*HbSWEET5a/5b*	MA	0.1044	0.2728	0.3828	2.03E‐05	543	142.305	400.695
*HbSWEET10e/10f*	MA	0.1524	0.1124	1.3556	0.2410	696	185.281	510.719
*HbSWEET15a/15b*	MA	0.0750	0.1856	0.4042	0.0001	690	179.627	510.373
*HbSWEET16b/16c*	MA	0.0584	0.3780	0.1544	6.24E‐18	684	186.783	497.217
*HbSWEET17a/17c*	MA	0.0806	0.2715	0.2970	7.67E‐08	615	157.587	457.413

Upon further examining the genomic locations, we found that some *SWEET* genes in the same clade were located adjacent to each other. For example, in Hevea*, HbSWEET4b* and *HbSWEET4c* were located on scaffold0371, *HbSWEET10a* and *HbSWEET10c* on scaffold1273, *HbSWEET10b, HbSWEET10d,* and *HbSWEET10f* on scaffold0491, *HbSWEET11* and *HbSWEET12* on scaffold0807, and *HbSWEET17a* and *HbSWEET17b* on scaffold0340. In *P. trichocarpa*,* PtSWEET1b, PtSWEET1c,* and *PtSWEET1d* were located adjacent to each other on chromosome 2, *PtSWEET3a* and *PtSWEET3c* on chromosome 15, *PtSWEET10a, PtSWEET10b, PtSWEET10c,* and *PtSWEET11* on chromosome 15, *PtSWEET16b, PtSWEET16c, PtSWEET17a,* and *PtSWEET17b* on chromosome 13. In *R. communis, RcSWEET4a* and *RcSWEET4d* were located on scaffold39822, and *RcSWEET10a, RcSWEET10b, RcSWEET11,* and *RcSWEET12* on scaffold30147. These adjacent gene pairs and clusters had apparently been derived from tandem duplication events.

### Structural organization of SWEET genes

**Figure 2:**
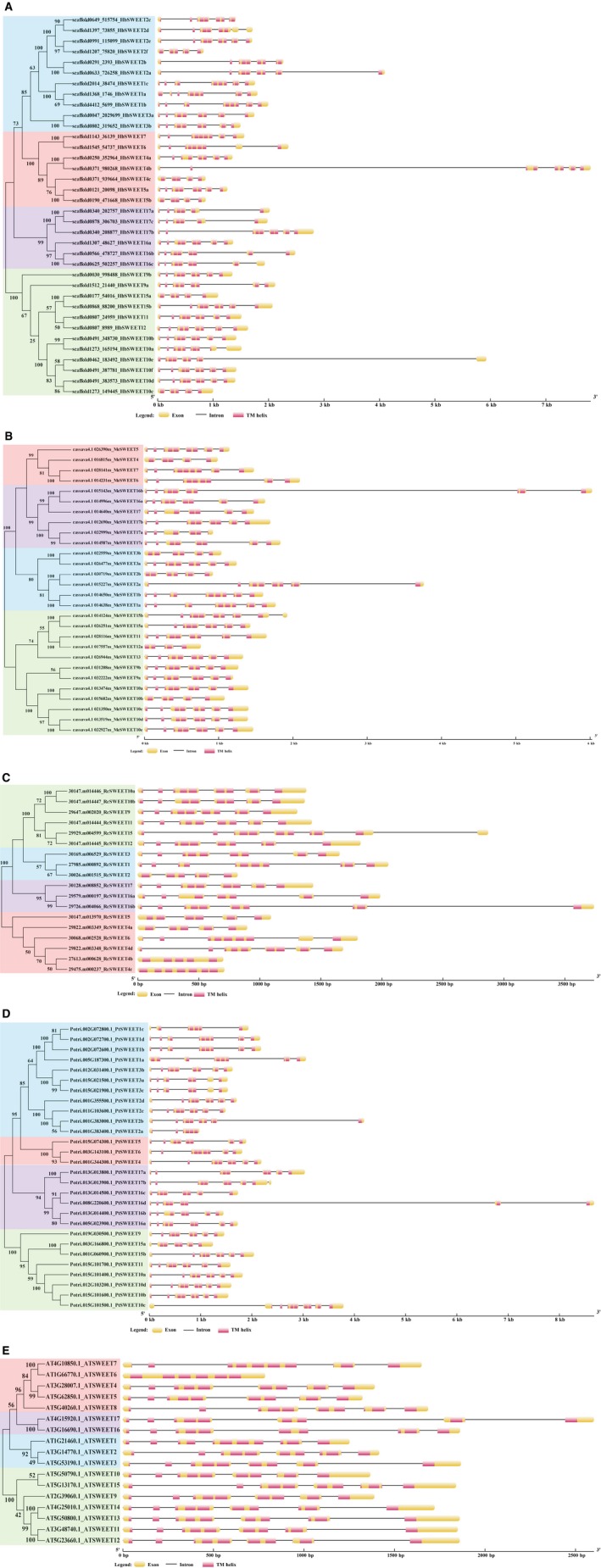
Structural organization of *SWEET* genes from *H. brasiliensis* and four other plant species. (A) to (E), structural organization of *SWEET* genes in *H. brasiliensis*,* M. esculenta*,* R. communis, P. trichocarpa*, and *A. thaliana,* respectively. Exons and introns are represented by boxes and black lines, respectively. The TM helix domain is represented by pink boxes. The sizes of exons and introns are proportional to their sequence lengths. Background shading: Clade 1, blue; Clade II, red; Clade III, Green; and Clade IV, Purple.

**Figure 3:**
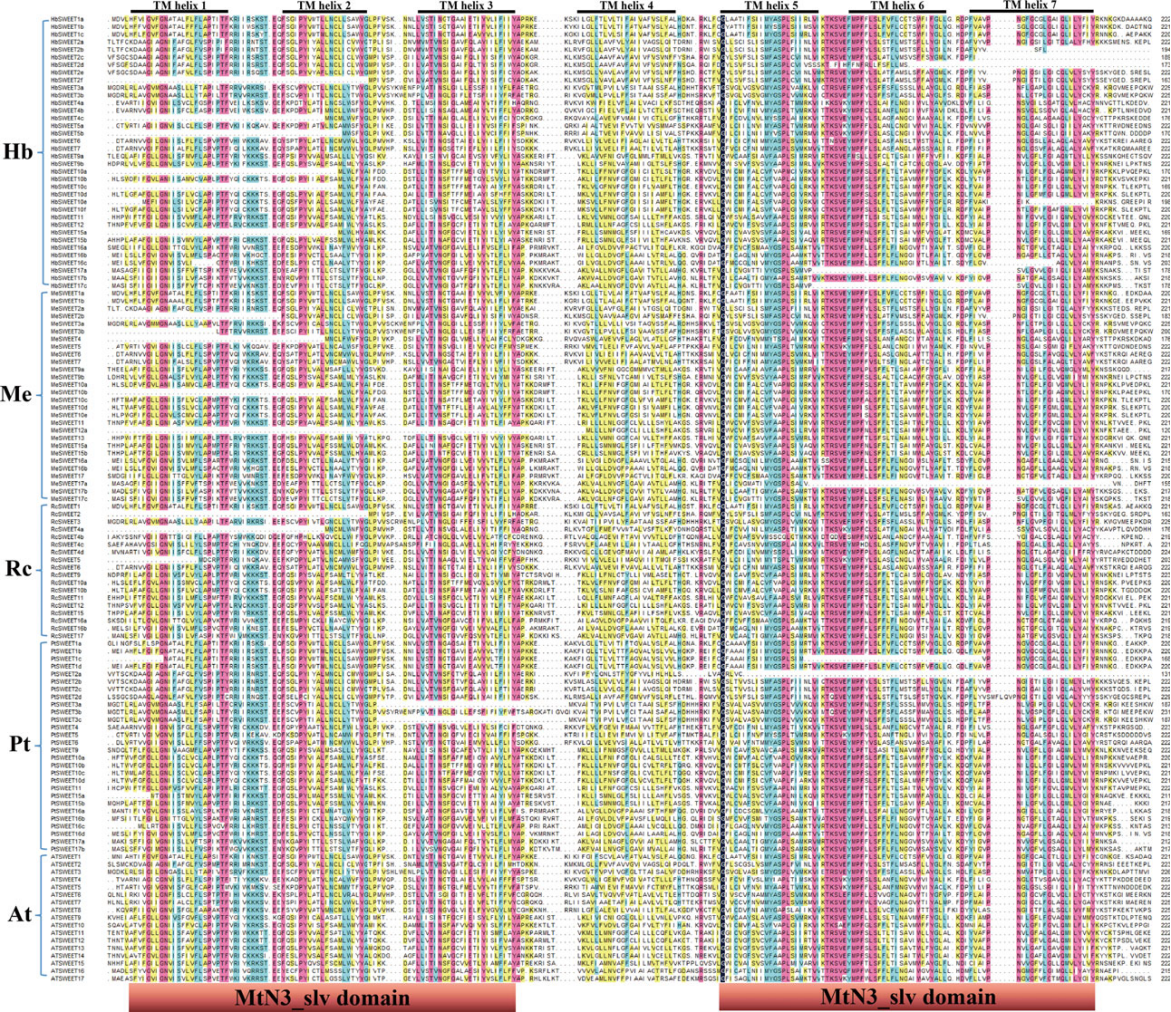
Multiple sequence alignment for the predicted amino acid sequences of the SWEET genes from *H. brasiliensis* and four other plant species. Sequence alignment was performed using DNAMAN 6.0 software (http://www.lynnon.com/). Identical amino acids are shaded, and gaps are indicated by dots.

The exon–intron structures of the 127 *SWEET* genes in five plant species were determined based on the predicted sequences. As shown in Fig. 2A, most Hevea *SWEET* members within the same groups share similar gene structures in terms of intron number, domain localization, and exon length. Although the lengths vary, introns are inserted into nearly the same locations of the gene ORFs. Most *SWEET* members contain 3–5 introns. Of the 36 members in Hevea, for example, 24 have 5 introns, 7 have 4 introns, and 5 have 3 introns (Fig. 2A, Table 1a). In the total of 127 SWEET genes among the five plant species, there were only three SWEET members with no introns, namely *RcSWEET4b*,* RcSWEET4c,* and *AtSWEET6,* all of which were clustered in Clade II (Fig. 2A‐E). Some SWEET members lacked exons at the 5′ end, such as *HbSEET2f, 4c, 5b, 15a, 10e,* and *10c, MeSWEET4, 3b, 2b, 12a,* and *10b, RcSWEET2, 5,* and *4a,* and *PtSWEET1c, 16c,* and *15a* (Fig. 2A‐E). Most SWEET members contain 4–7 TM helix domains, and 25 of the 127 members lost one to three of the seven TM helix typical of plant SWEETs (Fig. 3). In addition, the lengths of most *AtSWEET* and *RcSWEET* genes are shorter than those of the other plant *SWEET* genes, perhaps reflecting a relationship between gene length and genome size of a given species.

### Tissue expression of SWEET genes

To investigate the functions of *SWEET* genes*,* gene expression profiles in different tissues were analyzed by using Solexa sequencing data in Hevea*, M. esculenta*,* P. trichocarpa*, and *A. thaliana* (Tables S2, S3). Analysis of gene expression from the Sequence Read Archive (SRA), adopted in the present study, has limitations as the data were compiled from different sources where genetic differences in the tested tissues and dissimilarities in the experimental conditions can make comparisons difficult. Nonetheless, such an analysis provides a broad overview of the functionalities of the various Hevea *SWEETs* relative to their counterparts in other plant species. The results provide useful indicators as to which *SWEET* genes are most commonly expressed from among the numerous isoforms. These results would serve as a guide for future follow‐up research where more exacting methodologies can be employed.

**Figure 4:**
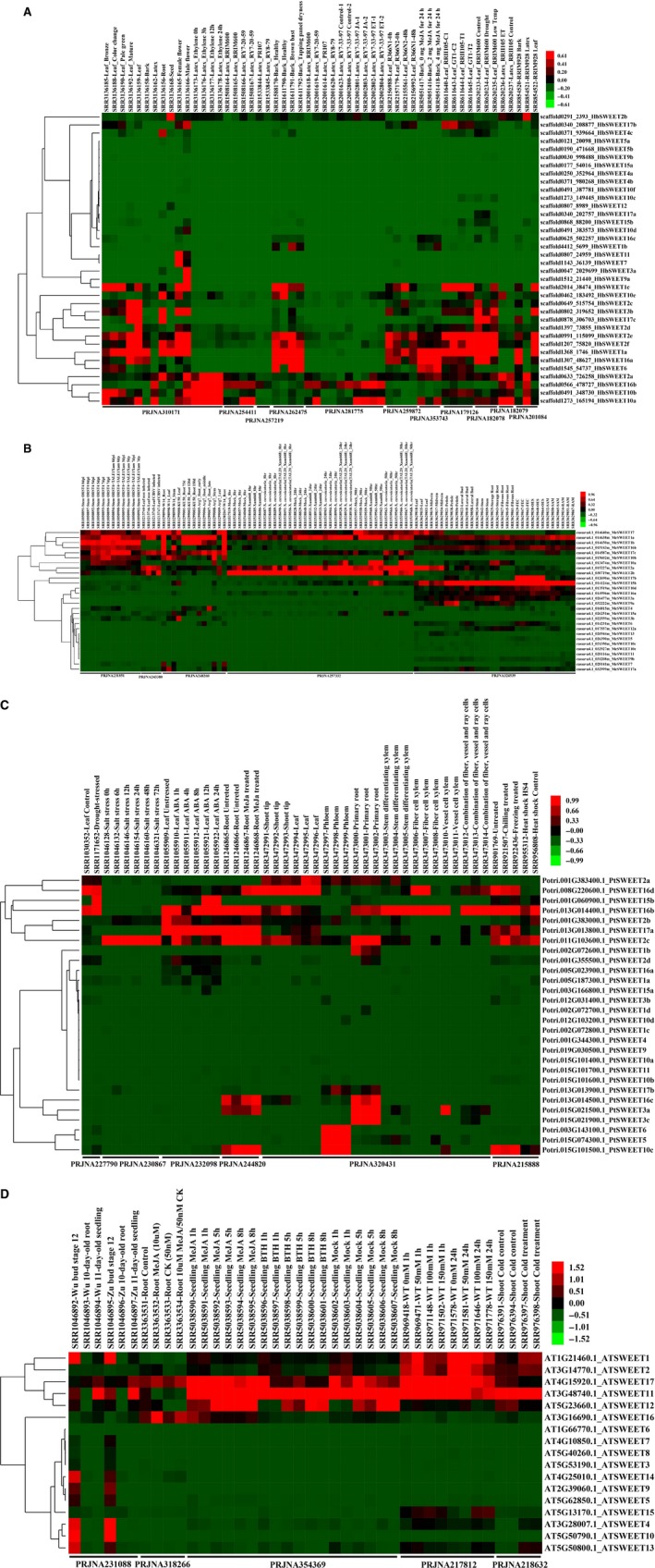
Expression analyses of the *SWEET* genes based on Solexa sequencing. (A), Hierarchical clustering and differential expression analysis of the *HbSWEET* genes in seven tissues (leaf, bark, latex, root, seed, female flower, and male flower), at four developmental stages of leaves (bronze, color change, pale‐green and mature), during ethephon treatment (0 h, 3 h, 12 h, and 24 h, PRJNA310171), latex (RRIM600 and RY7‐20‐59, PRJNA254411; clones PR107 and RY879, PRJNA257219), brown bast and tapping panel dryness (PRJNA262475), ET and JA treatment (PRJNA281775), Clone FX 3864 response to GCL012 (PRJNA259872), MeJA (PRJNA353743), *Corynespora cassiicola* tolerance (PRJNA179126), abiotic stress (drought, low temperature, PRJNA182078 and ethephon treatment, PRJNA182079), and tissues (leaf, bark, and latex, PRJNA201084); (B), Hierarchical clustering and differential expression analysis of the *MeSWEET* genes in different tissues (root, leaf, stem, PRJNA248260), infected by pathogenic Xanthomonas (PRJNA231851), CBSV virus (PRJNA243380), tissues (PRJNA324539), and bacterial blight pathogen (PRJNA257332); (C), Hierarchical clustering and differential expression analysis of the *PtSWEET* genes under ABA stimulation (0 h, 1 h, 4 h, 8 h, 12 h, and 24 h, PRJNA232098), methyl jasmonate stimulation (PRJNA244820), chilling, freezing, and heat shock (PRJNA207974, PRJNA215888), salinity stress (0 h, 6 h, 12 h, 24 h, and 72 h, PRJNA230867), tissues (PRJNA320431), drought stress (PRJNA227790); (D), Hierarchical clustering and differential expression analysis of the *AtSWEET* genes in different tissues (floral bud, root, seeding, PRJNA231088), MeJA or BTH (PRJNA354369), MeJA and CK (PRJNA318266), cold stress (PRJNA218632), salt stress (0 mm, 50 mm, 100 mm, 150 mm, PRJNA217812).

As shown in Fig. 4A, the expression levels of four *SWEET* genes (*HbSWEET1a*,* 2e*,* 2f*, and *3b*) in Hevea source leaves were significantly higher than those of the other members, while three *SWEET* genes (*HbSWEET1c*,* 10a*, and *10b*) were mainly expressed in sink leaves. In the other three plant species, *MeSWEET17, MeSWEET2a, MeSWEET15b, PtSWEET2a,* and *PtSWEET16d* were highly expressed in leaves and *AtSWEET11* was mainly expressed in seedling plants (11 days old) (Fig. 4B‐D). Some of the above‐mentioned SWEET members might be involved in phloem loading and leaf development. In Hevea bark where rubber‐producing laticifers reside, *HbSWEET1a* and *HbSWEET16a* showed a predominance expression, while in latex, the cytoplasm of laticifers, *HbSWEET2a, HbSWEET10a, HbSWEET10b and HbSWEET16b* were the predominant isoforms. These SWEET genes might play an important role in sugar transport between the laticifers and their neighboring bark tissues, and contribute to the regulation of sucrose concentrations in laticifers together with the sucrose transporter responsible for apoplasmic sucrose uptake of laticifers, HbSUT3 18, 19. In *A. thaliana, AtSWEET9* has been identified as a nectary‐specific sugar transporter 6. In Hevea*, HbSWEET9a* exhibited a male flower‐specific abundant expression and might have a similar function in nectary production as its *A. thaliana* orthologue, *AtSWEET9*. In addition, 14 other Hevea *SWEET* genes, viz. *HbSWEET1a, 1c, 2a, 2e, 2f, 3a, 3b, 7, 10a, 10b, 10e, 11, 16b,* and *17b*, were also expressed at high levels in flowers (Fig. 4A). No SRA expression data in flowers were found in the other three plant species. In Hevea roots, seven *SWEET* genes (*HbSWEET1a, 2a, 2f, 3b, 4c, 10e,* and *17c*) were abundantly expressed. In the other plant species, *MeSWEET10a, MeSWEET3a,* and *MeSWEET15b* were expressed at high levels in the storage roots of *M. esculenta*; their activities may be related to starch formation. Six *P. trichocarpa SWEET* genes (*PtSWEET2a, 2c, 3a, 3c, 16b,* and *16c*) were expressed at high levels in the roots. On the other hand, most of *A. thaliana SWEET* genes showed low or no expression in the roots. *PtSWEET16b* and *PtSWEET16d* exhibited high expression in xylem fiber cells that may be related to xylem formation. There were many *SWEET* genes showing universal expressions in most tissues examined. These included *HbSWEET1c, 10e, 2c, 3b, 17c, 2d, 2e, 2f, 1a, 16a, 6, 2a, 16b, 10b,* and *10a, MeSWEET17, 1a, 1b, 16b, 17c, 10b, 10a, 2a, 2b, 17b, 15b, 10d, 16a, 3a,* and *9a, PtSWEET2a, 16d, 15b, 16b, 2b, 17a, 2c, 16c, 3a,* and *10c,* and *AtSWEET1, 2, 17, 11, 12,* and *16*. Interestingly, isoforms of *SWEET2, 16,* and *17* were observed among the universally expressed SWEET genes in all plant species examined, which might represent the main evolutionary direction of *SWEET* genes in plants. As shown in Fig. 4A, transcripts of 11 *HbSWEET* genes (*HbSWEET4a, 4b, 5a, 5b, 9b, 10c, 10f, 12, 15a, 15b,* and *17a*) were barely detectable in almost all the tissues and all the treatments examined. Such genes comprise a large portion (~ 1/3) of the total *HbSWEET* gene family. This character seems to be shared by the *SWEET* gene families in other plant species. For example, similar expression patterns were observed for seven of 28 *SWEET* genes in *M. esculenta* (Fig. 4B), 12 of 28 in *P. trichocarpa* (Fig. 4C), and 4 of 17 in *A. thaliana* (Fig. 4D). This result suggests that the *SWEET* gene families in higher plants might have experienced an event of gene expansion followed by nonfunctionalization in the course of evolution. A similar phenomenon has been reported in our studies for the CDPK and CDPK‐related kinase gene families in Hevea 27. In addition, we found that most genes in Clade II have low or no expansion in all tissues examined in the four plant species.

### Expression profile of SWEET genes in response to hormone and stress treatments

Expression levels of *SWEET* genes in Hevea were also examined under various kinds of hormone and stress treatments. Ethephon, an ethylene generator, is widely used to stimulate latex production of the rubber tree, but the yield‐stimulating mechanisms are still poorly understood 15, 22. As shown in Fig. 4A, expressions of *HbSWEET10a* were obviously upregulated by ethephon treatment in latex. In addition, *HbSWEET10a* was the predominant SWEET isoform in latex, the expression of which was higher than any of the other SWEET members, suggesting its important role in sugar transport of laticifers. Expressions of *HbSWEET10a* and *HbSWEET2a* appeared to be regulated by methyl jasmonate (MeJA) although in differing manners. Expressions of *HbSWEET2c*,* HbSWEET2d,* and *HbSWEET3* were downregulated under drought treatment. Under low temperature treatment, expressions of *HbSWEET1c* were upregulated, whereas *HbSWEET2c, 2d, 16a,* and *17c* were downregulated. Expressions of Hevea *SWEET* genes were also regulated by other kinds of stress treatments. For example, the expressions of *HbSWEET1b*,* HbSWEET1c,* and *HbSWEET10e* were affected by tapping panel dryness, a complex physiological disorder affecting latex production severely 28; *HbSWEET17b* expressions were downregulated under the infection of *Corynespora cassiicola*, a fungal pathogen causing a leaf fall disease in Hevea 29.

The expression levels of *SWEET* genes in *M. esculenta*,* P. trichocarpa*, and *A. thaliana* were also examined when the plants were subjected to treatments of hormones and different stresses, including fungus infection, drought, and cold (Fig. 4B‐D). The expressions of six *MeSWEETs* (*MeSWEET1a, 10a, 10b, 15b, 17,* and *17c*) were affected by fungus infection. Expressions of *PtSWEET2b* and *PtSWEET16d* were induced by MeJA in roots. Expressions of *PtSWEET15b* were induced by drought and ABA (abscisic acid) treatments. In the model plant *A. thaliana* (Fig. 4D)*,* the expressions of *ATSWEET16* and *ATSWEET17* were upregulated by MeJA in roots. In seedlings, expressions of *ATSWEET12* were upregulated by MeJA, while those of *ATSWEET16* were downregulated. Under cold treatment, expressions of *ATSWEET1* and *ATSWEET2* were upregulated, while those of *ATSWEET16* and *ATSWEET17* were downregulated.

### Expression analyses of HbSWEET10a, HbSWEET16b, and HbSWEET1a based on qPCR

**Figure 5:**
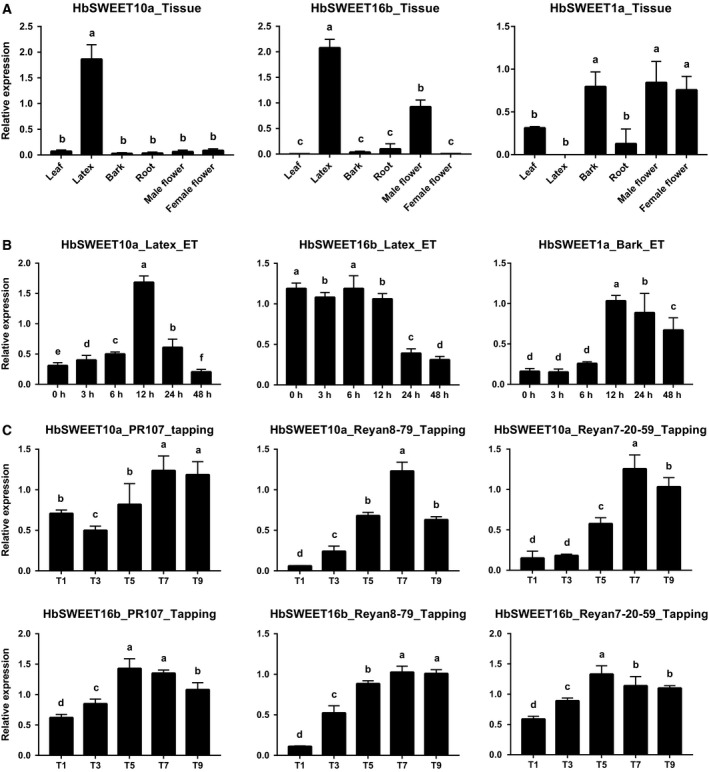
Expression analyses of *HbSWEET10a*,* HbSWEET16b,* and *HbSWEET1a* based on qPCR. (A), Expression of *HbSWEET10a*,* HbSWEET16b,* and *HbSWEET1a* transcripts in six tissues (leaf, latex, bark, root, male flower, and female flower). (B), Effect of Ethrel (2‐chloroethylphosphonic acid) treatment on *HbSWEET10a* and *HbSWEET16b* expression in latex, and *HbSWEET1a* expression in bark. (C), Transcript abundance of *HbSWEET10a* and *HbSWEET16b* in the first, third, fifth, seventh, and ninth tappings (T1, T3, T5, T7, and T9) on virgin Hevea trees of clones PR107, Reyan8‐79, and Reyan7‐20‐59. Values are means ± stdev of three biological replicates. Different letters indicate significant differences (Student's t‐test, *P* < 0.05).

Rubber is synthesized and stored in the cytoplasm (latex) of highly specialized cells called laticifers that are differentiated from the cambium and arranged in rings. To further examine the expression of *HbSWEET* genes in latex and bark, quantitative RT‐PCR (qPCR) analyses of *HbSWEET10a*,* HbSWEET16b,* and *HbSWEET1a* were performed. As shown in Fig. 5, the results from qPCR were in broad agreement with the sequencing‐based expression analyses. *HbSWEET10a* and *HbSWEET16b* were mainly expressed in latex; *HbSWEET1a* was mainly expressed in bark and flower (Fig. 5A). *HbSWEET10a* was obviously upregulated by ethephon treatment in latex, while *HbSWEET16b* was obviously downregulated after 24 hours of ethephon treatment in latex, which agrees well with the results based on RNA‐seq (Fig. 4A, 5B, Table S3‐1). We also further examined the expression of *HbSWEET1a* under ethephon treatment in bark, while *HbSWEET1a* was obviously upregulated (Fig. 5B).

The process of rubber harvesting, namely tapping, produces a conspicuous stimulating effect on latex production in virgin Hevea trees, and it has been partially ascribed to an enhanced sucrose uptake and sucrose catabolism in the laticifers 18, 20. As shown in Fig. 5C, *HbSWEET10a* and *HbSWEET16b* were obviously upregulated by tapping in three different clones PR107, Reyan7‐33‐97, and Reyan8‐79. All above results revealed that *HbSWEET10a, 16b, and 1a* might play an important role in sugar transport in laticifer and bark.

## Conclusion

In this study, a genome‐wide analysis of *SWEET* gene families was undertaken for the first time in Hevea, *M. esculenta,* and *R. communis*. *In silico* analysis of the Hevea genome database facilitated the identification of 36 *SWEET* genes. The phylogenetic analysis of 127 SWEETs from Hevea and four other plant species (*A. thaliana, P. trichocarpa, M. esculenta,* and *R. communis*) classified all these SWEETs into four major groups. Members within each group might have had common evolutionary origins as seen from the sharing of similar protein motifs, exon–intron structures, and basic molecular functions. Solexa sequencing analyses revealed that *SWEET2, 16,* and *17* were universally expressed in different tissues of all the plant species examined, possibly representing the main evolutionary direction of plant *SWEET* gene families. Extensive expressional analyses in different tissues and in response to various experimental treatments, including hormones, and biotic and abiotic stresses, identified multiple tissue‐specific SWEET isoforms and isoforms showing striking responses to some of the treatments in Hevea and three other plant species (*A. thaliana, P. trichocarpa,* and *M. esculenta*). These results indicate versatile roles of SWEETs in plant growth, development, and stress responses and provide a foundation for further functional investigation of the *SWEET* gene families in the plant kingdom.

## Materials and methods

### Database search for SWEET genes in *H. brasiliensis* and four other plant species

Sequences of *A. thaliana* and *P. trichocarpa* SWEET genes were downloaded from the *A. thaliana* Information Resource (http://www.Arabidopsis.org/) and GenBank (http://www.ncbi.nlm.nih.gov/genbank). The genome and protein sequences of *A. thaliana* 30, *P. trichocarpa* 31, *M. esculenta* 32, and *R. communis* 33 were downloaded from Phytozome v10 (http://www.phytozome.net/). The *H. brasiliensis* genome and transcriptome data were obtained from GenBank (http://www.ncbi.nlm.nih.gov/nuccore/448814761) 22. Local BLAST alignment was performed using published SWEET sequences from *A. thaliana* and *P. trichocarpa* as queries to search against the deduced proteome of each species for the candidate SWEETs from *H. brasiliensis*,* A. thaliana, P. trichocarpa, M. esculenta,* and *R. communis*. All putative candidates were manually verified with the InterProScan server (http://www.ebi.ac.uk/Tools/pfa/iprscan/) to confirm the presence of protein kinase and TM helix domains.

### Phylogenetic and gene structure analyses

Multiple alignments of the amino acid sequences of SWEETs from five species were performed using the Clustal X (version 1.83) program. The phylogenetic tree was constructed with MEGA6.0 34 by employing the neighbor‐joining (NJ) method with a bootstrap test for 1000 replicates. Exon–intron structures of the six species *SWEET* genes were analyzed by comparing the cDNA and their genomic DNA sequences through the web server GSDS 2.0 (http://gsds.cbi.pku.edu.cn/). The KaKs‐Calculator program (https://sourceforge.net/projects/kakscalculator2/) was used to calculate the nonsynonymous (Ka) and synonymous (Ks) substitutions in coding regions.

### Expression analysis based on Solexa sequencing

For Solexa sequencing‐based expression analyses, Sequence Read Archive (SRA) data were downloaded from the NCBI database (Table S2) 27. The sequences included those for *H. brasiliensis* (*C. cassiicola* tolerance, PRJNA179126; abiotic stress, PRJNA182078 and PRJNA182079; tissues, PRJNA201084 35; tissues, leaf development and ethephon treatment, PRJNA310171 22, 27; Clone FX 3864 response to GCL012, PRJNA259872; ET and JA treatment, PRJNA281775 36; brown bast and tapping panel dryness, PRJNA262475 28; Hevea clones PR107 and RY879, PRJNA257219 37; RRIM600 and RY7‐20‐59, PRJNA254411; MeJA, PRJNA353743); *M. esculenta* (*Xanthomonas* tolerance, PRJNA231851; CBSV virus infected, PRJNA243380; tissue, PRJNA248260; bacterial blight pathogen infected, PRJNA257332; tissue, PRJNA324539); *A. thaliana* (salt stress, PRJNA217812; tissues, PRJNA231088; cold stress, PRJNA218632; MeJA or BTH, PRJNA354369; MeJA and CK, PRJNA318266); *P. trichocarpa* (ABA stimulation, PRJNA232098; methyl jasmonate treatment, PRJNA244820; chilling, freezing and heat shock, PRJNA207974 and PRJNA215888; salinity stress, PRJNA230867; tissue, PRJNA320431; drought stress, PRJNA227790). Raw RNA‐seq reads were processed to trim terminal low‐quality bases and adapter sequences via an in‐house custom pipeline. The clean reads were then mapped to the genome using Bowtie2, and RSEM software was used for quantifying transcript abundance with default parameters 38.

### Quantitative reverse transcriptase PCR (qPCR)

To examine the expression of *HbSWEET10a*,* HbSWEET16b*, and *HbSWEET1a* in latex and bark, quantitative RT‐PCR (qPCR) was performed. Unless otherwise noted, Reyan7–33–97 (synonym for CATAS7–33–97 or RY3–33–97), Reyan8–79 (synonym for CATAS8–79 or RY8–79), and PR107 rubber trees (*H. brasiliensis*) selected for QPCR in this study were cultivated at the experimental plantation of the Rubber Research Institute of the Chinese Academy of Tropical Agricultural Sciences (Danzhou, Hainan, China). These trees were regularly tapped for latex collection in a half spiral pattern, every 3 days, without Ethrel stimulation (S/2, d/3). To study the tissue‐specific expression of *HbSWEET* genes, different tissues were collected from 10‐year‐old mature trees of clone Reyan7‐33‐97 that had been tapped for the last 2 years. The same type of tree was also used to examine the effect of Ethrel on expression. To analyze the effect of tapping on *HbSWEET* genes expression, 8‐year‐old mature virgin (never tapped) trees of clones PR107, Reyan8‐79, and Reyan7‐20‐59 were selected. The reaction was performed using the Light Cycler 2.0 system (Roche Diagnostics, Penzberg, Germany) using SYBR Green premix kit (TaKaRa) according to the manufacturer's instructions. The primer pairs used for the *HbSWEET* genes were 5′‐CTGCACATGC AACTCACTCACA‐3′ (F) and 5′‐CATCGGGTGGTGTAATGCTCT‐3′(R) (*HbSWEET10a*), 5′‐GT TCGCCTCTTGCTGCCA‐3′ (F) and 5′‐AAGTCCAAATCCCTCCGTTCA‐3′ (R) (*HbSWEET16b*), 5′‐TCTCCTTTCCGCCTGGTATG‐3′ (F) and 5′‐GCTCTTCTCCTTCCTTGGTGC‐3′ (R) (*HbSWEET1a*). For genes as internal control, *YLS8* was used in gene expression analyses in the latex responding to tapping and Ethrel treatment, *RH2b* was used for tissue expression as recommended by Li *et al*. (2011) 39. The details for experimental manipulations and data analysis were as described by Tang *et al*. (2010) 18.

## Authors contributions

CRT conceived and designed the experiments. JLS, XHX, and JYQ performed the experiments. XHX and YJF analyzed the data. JLS, XHX, and CRT wrote the manuscript. All authors read and approved the final manuscript.

## Supporting information


**Table S1.** SWEET Accessions.

**Table S2.** Basic information for Solexa sequencing data of *Hevea brasiliensis* and three other plant species.

**Table S3.** RNA‐seq analysis of the expressions of SWEET genes in *Hevea brasiliensis* and three other plant species.
