# Supplementary material for: The SWEET gene family in Hevea brasiliensis – its evolution and expression compared with four other plant species
Source: FEBS Open Bio. 2017 Oct 30;7(12):1943–59. doi: 10.1002/2211-5463.12332 (PMC5715295; doi:10.1002/2211-5463.12332)
Supplement: Supplementary file 1 — Table S1. SWEET Accessions. [file FEB4-7-1943-s001.pdf]

Supplementary Table S1. SWEET Accessions.

Hevea brasiliensis	*Manihot esculenta*	*Ricicus communis*	*Populus trichocarpa*	*Arabidopsis thaliana*
HbSWEET1a-scaffold1368_1746	MeSWEET1a-cassava4.1_014638m	RcSWEET1-27985.m000892	PtSWEET1a-Potri.005G187300.1	ATSWEET1-AT1G21460.1
HbSWEET1b-scaffold4412_5699	MeSWEET1b-cassava4.1_014650m	RcSWEET2-30026.m001515	PtSWEET1b-Potri.002G072600.1	ATSWEET2-AT3G14770.1
HbSWEET1c-scaffold2014_38474	MeSWEET2a-cassava4.1_015227m	RcSWEET3-30169.m006529	PtSWEET1c-Potri.002G072800.1	ATSWEET3-AT5G53190.1
HbSWEET2a-scaffold0633_726258	MeSWEET2b-cassava4.1_030719m	RcSWEET4a-29822.m003349	PtSWEET1d-Potri.002G072700.1	ATSWEET4-AT3G28007.1
HbSWEET2b-scaffold0291_2393	MeSWEET3a-cassava4.1_026477m	RcSWEET4b-27613.m000628	PtSWEET2a-Potri.001G383400.1	ATSWEET5-AT5G62850.1
HbSWEET2c-scaffold0649_515754	MeSWEET3b-cassava4.1_022559m	RcSWEET4c-29475.m000237	PtSWEET2b-Potri.001G383000.1	ATSWEET6-AT1G66770.1
HbSWEET2d-scaffold1397_73855	MeSWEET4-cassava4.1_016815m	RcSWEET4d-29822.m003348	PtSWEET2c-Potri.011G103600.1	ATSWEET7-AT4G10850.1
HbSWEET2e-scaffold0991_115099	MeSWEET5-cassava4.1_026390m	RcSWEET5-30147.m013970	PtSWEET2d-Potri.001G355500.1	ATSWEET8-AT5G40260.1
HbSWEET2f-scaffold1207_75820	MeSWEET6-cassava4.1_014231m	RcSWEET6-30068.m002528	PtSWEET3a-Potri.015G021500.1	ATSWEET9-AT2G39060.1
HbSWEET3a-scaffold0047_2029699	MeSWEET7-cassava4.1_028141m	RcSWEET9-29647.m002020	PtSWEET3b-Potri.012G031400.1	ATSWEET10-AT5G50790.1
HbSWEET3b-scaffold0802_319652	MeSWEET9a-cassava4.1_032222m	RcSWEET10a-30147.m014446	PtSWEET3c-Potri.015G021900.1	ATSWEET11-AT3G48740.1
HbSWEET4a-scaffold0250_352964	MeSWEET9b-cassava4.1_031208m	RcSWEET10b-30147.m014447	PtSWEET4-Potri.001G344300.1	ATSWEET12-AT5G23660.1
HbSWEET4b-scaffold0371_980268	MeSWEET10a-cassava4.1_013474m	RcSWEET11-30147.m014444	PtSWEET5-Potri.015G074300.1	ATSWEET13-AT5G50800.1
HbSWEET4c-scaffold0371_939664	MeSWEET10b-cassava4.1_015602m	RcSWEET12-30147.m014445	PtSWEET6-Potri.003G143100.1	ATSWEET14-AT4G25010.1
HbSWEET5a-scaffold0121_20098	MeSWEET10c-cassava4.1_021350m	RcSWEET15-29929.m004599	PtSWEET9-Potri.019G030500.1	ATSWEET15-AT5G13170.1
HbSWEET5b-scaffold0190_471668	MeSWEET10d-cassava4.1_013519m	RcSWEET16a-29579.m000197	PtSWEET10a-Potri.015G101400.1	ATSWEET16-AT3G16690.1
HbSWEET6-scaffold1545_54737	MeSWEET10e-cassava4.1_032927m	RcSWEET16b-29726.m004066	PtSWEET10b-Potri.015G101600.1	ATSWEET17-AT4G15920.1
HbSWEET7-scaffold1143_36139	MeSWEET11-cassava4.1_028116m	RcSWEET17-30128.m008852	PtSWEET10c-Potri.015G101500.1	
HbSWEET9a-scaffold1512_21440	MeSWEET12a-cassava4.1_017557m		PtSWEET10d-Potri.012G103200.1	
HbSWEET9b-scaffold0030_998488	MeSWEET13-cassava4.1_026944m		PtSWEET11-Potri.015G101700.1	
HbSWEET10a-scaffold1273_165194	MeSWEET15a-cassava4.1_026251m		PtSWEET15a-Potri.003G166800.1	
HbSWEET10b-scaffold0491_348730	MeSWEET15b-cassava4.1_014124m		PtSWEET15b-Potri.001G060900.1	
HbSWEET10c-scaffold1273_149445	MeSWEET16a-cassava4.1_014996m		PtSWEET16a-Potri.005G023900.1	
HbSWEET10d-scaffold0491_383573	MeSWEET16b-cassava4.1_015143m		PtSWEET16b-Potri.013G014400.1	
HbSWEET10e-scaffold0462_183492	MeSWEET17-cassava4.1_014640m		PtSWEET16c-Potri.013G014500.1	
HbSWEET10f-scaffold0491_387781	MeSWEET17a-cassava4.1_032999m		PtSWEET16d-Potri.008G220600.1	
HbSWEET11-scaffold0807_24959	MeSWEET17b-cassava4.1_012690m		PtSWEET17a-Potri.013G013800.1	
HbSWEET12-scaffold0807_8989	MeSWEET17c-cassava4.1_014587m		PtSWEET17b-Potri.013G013900.1	
HbSWEET15a-scaffold0177_54016				
HbSWEET15b-scaffold0868_88200				
HbSWEET16a-scaffold1307_48627				
HbSWEET16b-scaffold0566_478727				
HbSWEET16c-scaffold0625_502257				
HbSWEET17a-scaffold0340_202757				
HbSWEET17b-scaffold0340_208877				
HbSWEET17c-scaffold0878_306703				
